# Dual Crisscross Attention Module for Road Extraction from Remote Sensing Images

**DOI:** 10.3390/s21206873

**Published:** 2021-10-16

**Authors:** Chuan Chen, Huilin Zhao, Wei Cui, Xin He

**Affiliations:** 1 TUM Department of Aerospace and Geodesy, Technical University of Munich, 80333 Munich, Germany; chuan.chen@tum.de; 2 School of Resources and Environmental Engineering, Wuhan University of Technology, Wuhan 430070, China; zhaohl2016@whut.edu.cn (H.Z.); 2962575697@whut.edu.cn (X.H.)

**Keywords:** remote sensing, semantic segmentation, road extraction, attention mechanism, geometric information, directionality

## Abstract

Traditional pixel-based semantic segmentation methods for road extraction take each pixel as the recognition unit. Therefore, they are constrained by the restricted receptive field, in which pixels do not receive global road information. These phenomena greatly affect the accuracy of road extraction. To improve the limited receptive field, a non-local neural network is generated to let each pixel receive global information. However, its spatial complexity is enormous, and this method will lead to considerable information redundancy in road extraction. To optimize the spatial complexity, the Crisscross Network (CCNet), with a crisscross shaped attention area, is applied. The key aspect of CCNet is the Crisscross Attention (CCA) module. Compared with non-local neural networks, CCNet can let each pixel only perceive the correlation information from horizontal and vertical directions. However, when using CCNet in road extraction of remote sensing (RS) images, the directionality of its attention area is insufficient, which is restricted to the horizontal and vertical direction. Due to the recurrent mechanism, the similarity of some pixel pairs in oblique directions cannot be calculated correctly and will be intensely dilated. To address the above problems, we propose a special attention module called the Dual Crisscross Attention (DCCA) module for road extraction, which consists of the CCA module, Rotated Crisscross Attention (RCCA) module and Self-adaptive Attention Fusion (SAF) module. The DCCA module is embedded into the Dual Crisscross Network (DCNet). In the CCA module and RCCA module, the similarities of pixel pairs are represented by an energy map. In order to remove the influence from the heterogeneous part, a heterogeneous filter function (HFF) is used to filter the energy map. Then the SAF module can distribute the weights of the CCA module and RCCA module according to the actual road shape. The DCCA module output is the fusion of the CCA module and RCCA module with the help of the SAF module, which can let pixels perceive local information and eight-direction non-local information. The geometric information of roads improves the accuracy of road extraction. The experimental results show that DCNet with the DCCA module improves the road IOU by 4.66% compared to CCNet with a single CCA module and 3.47% compared to CCNet with a single RCCA module.

## 1. Introduction

Road extraction has become a popular topic as a branch subject of semantic segmentation, and many complicated deep learning methods have been developed [1,2,3] and improved continuously to pursue a higher accuracy.

Some researchers have focused on the loss function. He et al. [3] proposed an encoder-decoder network model with a special loss function, which was optimized by road structure constraints. This method improved the detection of road coherence to a certain extent. In addition, many deep learning models based on the fully convolution network (FCN) have been proposed [1,4]. Some researchers have analysed the effective area of the receptive field in traditional convolutional neural networks (CNNs). The active receptive field presents a Gaussian distribution, which will lead to difficulties for each pixel in obtaining contextual information [5]. These road extraction methods were generally at the pixel level, which would lead to local receptive area limits; therefore, each pixel will not receive enough global information [6]. However, the roads in remote sensing images have multidirectional characteristics. Due to the lack of perception of global information, the geometric information of the road has not been fully utilized.

To solve the poor receptive field problem, many constructions have been proposed to expand the receptive field range. Wang et al. [2] proposed the non-local neural network, which calculates the feature similarity between each pixel and other pixels on the feature map. In this way, the global information can be integrated into the centre pixel. However, this method needs to calculate the relationship of each pixel pair on the feature map, which will lead to a high computational complexity. In specific semantic segmentation problems, it will generate information redundancy. Some researchers have focused on the convolution process and produced dilated convolutions to enlarge the receptive field [7]. However, the dilated convolution has some flaws. When using dilated convolutions, the effective receptive field of the pixels at the edge of the feature map is quite different from that of the pixels at the centre. The uncertainty of the effective receptive field will make it untargeted to solve semantic segmentation problems.

Following the idea of reducing computational cost and information redundancy, Huang et al. [8] generated the CCNet by using a crisscross shape attention module, which can expand the receptive field in the vertical and horizontal directions at the same time. Using a recurrent mechanism, CCNet can let each pixel receive global information with a relatively low computational cost. However, this mechanism will also lead to a recurrent dilemma, which means that the similarity calculation of two pixels will be influenced by the intermediate pixel. In extreme cases, the heterogeneity of the intermediate nodes will reduce the similarity of homogeneous pixels in the oblique direction.

**Figure 1:**
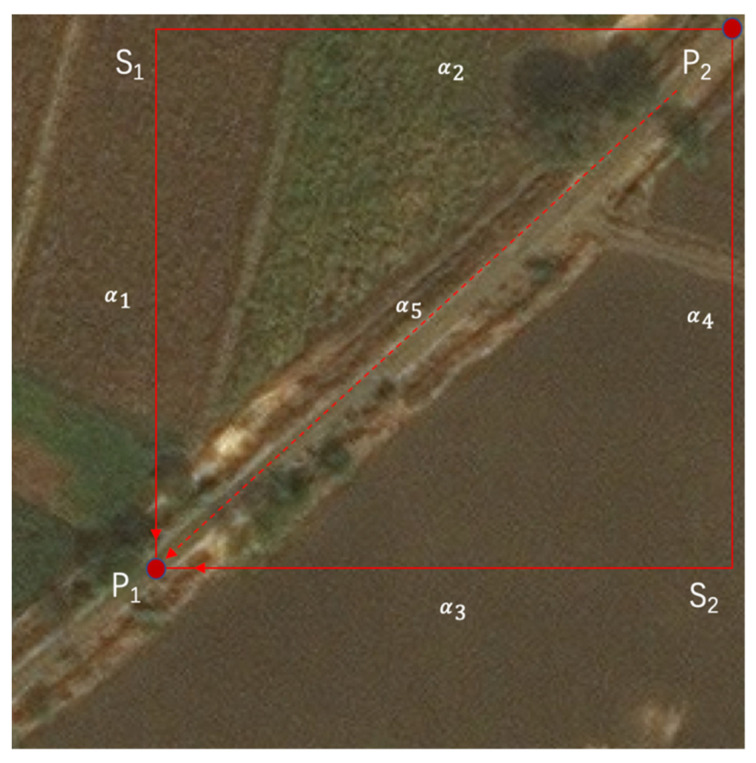
Recurrent dilemma.

For example, there are two pixels P1 and P2 in the Figure 1:

P1 and P2 are two points on the road, while S1 and S2 are two points on the non-road part. S1 and S2 are in the vertical and horizontal directions of P1 and P2. Using a recurrent mechanism [8], the relation between P1 and P2 needs to contain the transitions in S1 and S2, which can be described as P1→S1→P2 and P1→S2→P2. In the similarity calculation, by processing the feature vector, the similarity of P1 and S1 is $\alpha_{1}$. The similarities of S1 and P2, P1 and S2, S1 and P2 are $\alpha_{2},\;\alpha_{3},\;$ and $\alpha_{4}$, respectively. The similarity of P1 and P2, which is directly calculated by the feature vectors, is $\alpha_{5}$. Due to the heterogeneity of S1 and P1, $\alpha_{1}$ is relatively smaller than $\alpha_{5}$. Moreover, $\alpha_{2},\;\alpha_{3},\;$ and $\alpha_{4}$ are also relatively smaller than $\alpha_{5}$ for the heterogeneity of corresponding points. In the recurrent mechanism, the similarity of P1 and P2 will be $\alpha_{1}\times \alpha_{2}+\alpha_{3}\times \alpha_{4}$, which can be regarded as the second-order small amount compared to $\alpha_{5}$, which is why the recurrent mechanism will lead to the distortion of similarity. In the methodology section, we will illustrate this in detail by the formula.

Consequently, the key shortcoming of CCNet is that the crisscross shape attention module has poor directionality when extracting multidirectional objects, especially roads. Considering the multidirectionality of roads, we add two extra attention areas, with one line in the 45° direction and another line in the 135° direction, to let each pixel receive more directional information. These two lines are called the RCCA module. The CCA module and RCCA module can let each pixel receive contextual information from eight directions. The computational cost of RCCA module is two-thirds of the computational cost of CCA module.

In the attention process, the generation of weights is an important step, where the activation function is often used. Some researchers choose the sigmoid function to output the final weights in the spatial attention module [9]. The activation function can be regarded as a selection of the feature. According to the requirements of semantic segmentation work, we proposed an HFF, which can largely reduce the influence of the heterogeneous part in the attention area.

Then we proposed a SAF module that can distribute the weight of the CCA module and RCCA module according to the specific direction of roads in images. The combination of the SAF module, CCA module and RCCA module is called the DCCA module. The computational cost of DCCA module is five-thirds of the computational cost of CCA module.

The innovations are based on the following aspects.

In the road extraction area, we proposed the RCCA module, which expands the attention area to pixels in the oblique direction for the first time. Consequently, it can solve the recurrent dilemma in DCCA module was designed by distributing the weight of the CCA module and RCCA module according to the specific road shape. The DCCA module can let each pixel receive contextual information from eight directions and not only local pixel information from convolutions. Therefore, it can be regarded as the combination of eight directional nonlocal attention mechanisms and the local convolution mechanism.

A heterogeneous filter function is created to suppress the influence of heterogeneous regions in the attention process. Processed by the heterogeneous filter function, each pixel can largely receive contextual information from homogeneous areas, which can promote the extraction accuracy.

In the following sections, we will introduce the related work, methodology, experiment, and conclusion. In the related work section, we will give an overview of the research content related to our work. Then, in the methodology section, we will give a detailed description of the implementation method of the DCCA module and the grafted network. In the experimental section, we will show the advantages of the DCCA module on the attention of road directionality through experimental analysis.

## 2. Related Work

### 2.1. Semantic Segmentation Based on Deep Learning

With the advent of LeNet in 1998, convolutional neural networks (CNNs) began to be widely used in image information processing [10]. The main elements of CNNs were also determined at this time, including convolutional layer, pooling layer, fully connected layer, etc. Researchers designed AlexNet and let people see the potential of CNN in image processing for the first time in the ImageNet competition in 2012 [11]. Since then, the image processing capabilities have been transferred to semantic segmentation, and various networks, including different architectures, exist in the semantic segmentation stage.

The deep learning algorithm of semantic segmentation affixes class labels to each pixel. That is, the general workflow can be regarded as the interpretation of the pixels. After years of development, there are many prominent semantic segmentation neural networks, such as FCN [4], U-Net [12], SegNet [13], and DeepLab [14]. However, FCNs and other networks based on the CNN structure limit the range of the receptive field and can only obtain short-range context information. Although the traditional deep convolutional neural network obtains global context information by superimposing multiple convolutions, related studies have shown that the actual perception range of this method is smaller than the theoretical expected value [6]. Some researchers have found that this kind of receptive field has an irregular Gaussian distribution around the central pixel [5]. Consequently, the long-distance dependency relation inside the sample cannot be processed properly. To address this problem, Chen et al. proposed the Atrous Spatial Pyramid Pooling (ASPP) module with multiscale dilated convolution to integrate context information [14,15,16]. On this basis, Zhao et al. further proposed Pyramid Scene Parsing Network (PSPNet) with a pyramid pooling module to capture contextual information [17]. These kinds of methods based on dilated convolution still have the deflection that they obtain information from a small number of surrounding points and cannot form a dense context information structure. At the same time, methods based on pooling lose too much spatial information and thus cannot effectively meet the pixel-by-pixel classification requirements of semantic segmentation. To effectively obtain the global context information of the pixel, PSPNet learns to summarize the contextual information of each pixel by the predicting attention map [18]. A non-local network uses a self-attention mechanism to enable each pixel to perceive the features of pixels at all other locations, which can produce more powerful pixel-level characterization capabilities [2].

With the advancement of the attention mechanism in the application of semantic segmentation, crisscross attention [8], a very prominent attention method, was proposed. Crisscross attention proposed measures in view of the large amount of calculation and low efficiency of non-local networks by using a crisscross shape attention module, which can expand the receptive field in the vertical and horizontal directions at the same time. However, it also has a certain problem that the attention directionality is limited. When solving the semantic segmentation problem, an attention area, which can help centre pixels obtain contextual information from eight directions with automatic weights, is more appropriate to interpret objects with complex directionality.

### 2.2. Attention Mechanism and Its Implementation in CNN

The attention mechanism has been widely used in natural language processing and computer vision [19,20]. The attention mechanism in computer vision simulates the human recognition process of an image, which means that the perception system does not process the entire scene at once but puts attention to certain specific parts to obtain the information with high priority. The priority of these parts is selected by preset preferences in the human brain, such as for colour, shape, and characteristics.

Some researchers have implemented attention mechanisms in image captioning tasks. They proposed soft attention and hard attention architectures with a visualization method of the attention area. Researchers at Google [21] first proposed the transformer structure based on self-attention and the multihead self-attention structure. The key, query, and value of self-attention are output by sequence-to-sequence type, which becomes the basis of subsequent attention research. Wang et al. [2] proposed a nonlocal neural network, which can remove the local receptive field limitation of convolution and capture global information effectively. Hu et al. proposed a channel attention mechanism, using global average pooling and full connection to focus on the attention weight of channel feature extraction [22]. Some researchers have proposed the convolutional block attention module (CBAM), which can carry out spatial attention and channel attention to the feature map at the same time [9]. The embed type of the CBAM is series connection. Based on this, Fu et al. used parallel connections to embed spatial attention and channel attention in neural networks [23].

These attention methods can be summarized as self-attention families. Generally, the self-attention mechanism can capture the spatial dependence of any two positions in the feature map and obtain global context information, thereby greatly improving the performance of the semantic segmentation network [24,25].

### 2.3. Attention Mechanism and Its Implementation in CNN

In recent years, a variety of methods have been proposed to extract roads from remote sensing images. These methods can be generally divided into two categories: road area extraction and road centerline extraction. Road area extraction [26,27,28,29,30,31] can generate pixel-level markers of roads, and the purpose of road centerline extraction [32,33,34,35] is to detect the skeleton of the road.

Zhang et al. first applied a support vector machine (SVM) to the road extraction of remote sensing images based on edge detection [36]. Song et al. proposed a method using shape index features and support vector machines (SVMs), which put geometric features into consideration for the first time [37]. Based on this, researchers use salient features to design a multilevel framework, which can extract roads from high-resolution multispectral images [38].

With the development of deep learning, road extraction methods based on deep learning have shown better performance than non-deep learning methods. Researchers have proposed a method to detect road areas from high-resolution aerial images using restricted Boltzmann machines, which first implemented deep learning tools [27]. Compared to this method, researchers have used CNNs to extract roads and buildings and obtain better results [30]. Alvarez et al. [39] proposed an automatic road extraction method based on U-Net. Zhong et al. [40] proposed a semantic segmentation neural network that combines the advantages of residual learning and U-Net for road extraction, which simplified training and achieved better results with fewer parameters.

Zhang et al. [41] used D-Link-Net and DenseNet for high-resolution satellite image road extraction. Based on this, Peng et al. [42] proposed a multiscale enhanced road detection framework (Dense-U-Net) based on densely connected convolutional networks (Dense-Net) and U-Net, which can effectively perform feature learning and retain finer spatial details.

Generally, the method of road extraction based on deep learning focuses more on the use of road features. These methods lack attention to geometric information, such as directionality. The DCCA module fills this vacancy.

## 3. Methodology

In the methodology section, we will first provide a description of the framework. Then, we will introduce the implementation details of the CCA module and the grafted network. The design of the RCCA module will be described in the RCCA part. In heterogeneous filter function and output part, the HFF and SAF module will be introduced.

### 3.1. Framework

**Figure 2:**
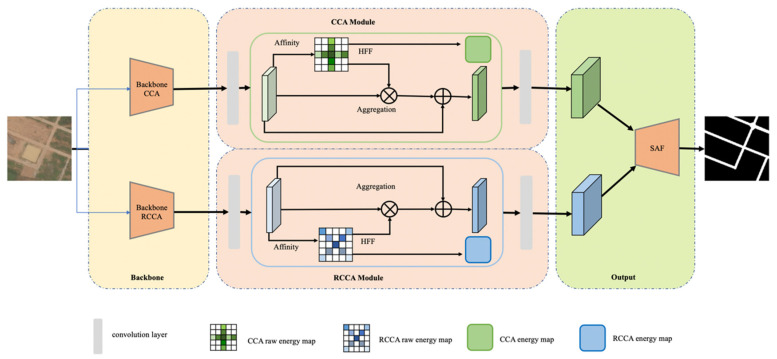
Network Structure.

The neural network is based on the DCCA module, which is called DCNet. DCNet consists of three main parts: backbone part, attention part and output part. The backbone part extracts the feature by traditional convolutions, which supports obtaining the correlation between pixels in different directions. The attention part consists of the CCA module and RCCA module, which can constitute an eight-direction nonlocal attention mechanism. HFF plays an important role in the energy process inside the attention part. In the output part, the SAF module can distribute the weights of these two modules according to the energy distribution in the sampling area and fuse the correlation information from eight directions. Considering the use of local 3 × 3 convolutions, the output part can realize the combined acquisition of 3 × 3 local information and eight directions of nonlocal information. The network structure is shown in the Figure 2.

#### 3.1.1. Backbone

**Figure 3:**
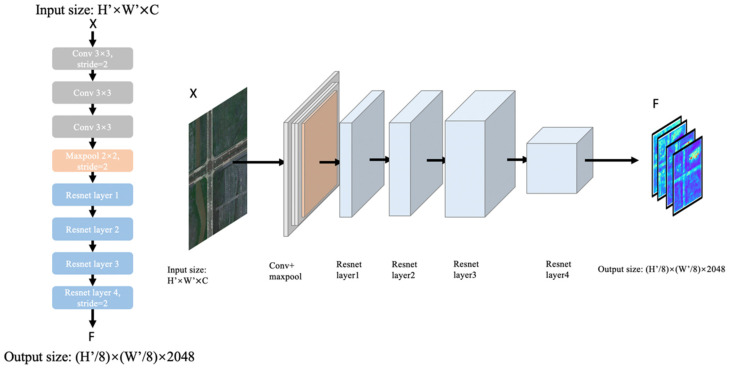
The backbone part.

The backbone part consists of two parallel backbones that are used for feature extraction, which are based on the residual network. Each backbone is shown in the Figure 3.

X ∈ ${\mathbb{R}}^{H\prime \times W\prime \times C}$ is input at the beginning of the network. The first process is three convolutions and one max-pooling, and then it comes to the feature extraction based on ResNet101. After three downsamplings, the output *F* ∈ ${\mathbb{R}}^{(H\prime /8)\times (W\prime /8)\times C}$ is generated.

This process can be expressed by the following formula:

(1) $$\begin{array}{c} F=\{\begin{array}{c} \;\;\;\;f_{CCA}\left(\;X\;\right),\;\;\;\;\;CCA\;Backbone\\ \;\;\;\;\;f_{RCCA}\left(\;X\;\right),\;\;\;\;RCCA\;Backbone \end{array} \end{array}$$

#### 3.1.2. The Implementation of CCA Module

**Figure 4:**
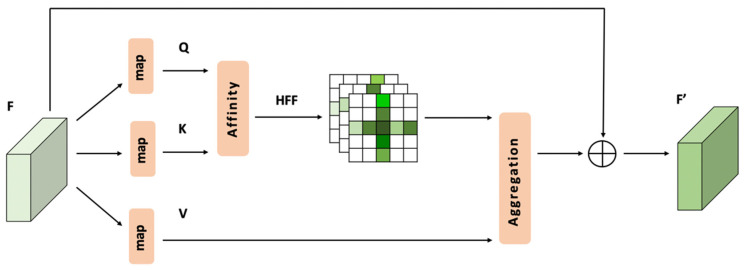
CCA module.

The implementation of the CCA is generally simple compared to that of the RCCA. Therefore, it will be shown firstly in Figure 4.

This attention method is from Huang et al. [8], which is used as the CCA module as part of the DCCA module.

First, we represent the dimension of *F* ∈ ${\mathbb{R}}^{(H\prime /8)\times (W\prime /8)\times \;C}$ as *F* ∈ ${\mathbb{R}}^{H\times W\times C}$.

As shown in the Figure 4, the local feature map *F*, the output of the backbone part, is mapped to *K* ∈ ${\mathbb{R}}^{H\times W\times C}$, *Q* ∈ ${\mathbb{R}}^{H\times W\times C}$ and *V* ∈ ${\mathbb{R}}^{H\times W\times C}$.

(2) $$\begin{array}{c} K,Q,V=F \end{array}$$

Then, an affinity process is used to process *K* ∈ ${\mathbb{R}}^{H\times W\times C}$ and *Q* ∈ ${\mathbb{R}}^{H\times W\times C}$. For each position (i,j), the centre point channel vector $Q_{ij}$ is multiplied by the channel vectors of crisscross attention area $K_{ij}^{\Phi }$.

(3) $$K_{ij}^{\Phi }=K_{ij}\{(x^{I},y^{I})\;|x^{I}=i,\;y^{I}\epsilon \;[1,\cdots ,W]\}\;\cup \;K_{ij}\left\{\left(x^{II},\;y^{II}\right)\;|\;x^{II}\epsilon \;\left[1,\cdots ,H\right],\;y^{II}=j\right\}\;\;\begin{array}{c} i\;\epsilon \;\left[1,\cdots ,H\right],\;j\;\epsilon \;\left[1,\cdots ,W\;\right] \end{array}$$

where $\left\{\left(x^{I},\;y^{I}\right)|\ldots \right\}$ and $\left\{\left(x^{II},\;y^{II}\right)|\ldots \right\}$ are two point groups that represent the horizontal line and vertical line in the crisscross area. The output of the affinity process is called raw energy map D ∈ ${\mathbb{R}}^{(H+W-1)\times H\times W}$.

$$D_{ij}=Q_{ij}\;\cdot \;K_{ij}^{\Phi }\;i\epsilon \left[1,\cdots ,H\right],j\epsilon \left[1,\cdots ,W\right]$$

For each position (i,j) in the spatial dimension of D ∈ ${\mathbb{R}}^{(H+W-1)\times H\times W}$, we use HFF to process the raw energy vector $D_{ij}$ ∈ ${\mathbb{R}}^{H+W-1}$. The result is labelled $A_{ij}$ ∈ ${\mathbb{R}}^{H+W-1}$, which is the energy vector in position (i,j) of the energy map A ∈ ${\mathbb{R}}^{(H+W-1)\times H\times W}$.

$$A_{ij}=HFF\left(\;D_{ij}\;\right)$$

For each position (i,j), an aggregation process is used to process the channel vector $A_{ij}$ and the channel vectors of crisscross attention area $V_{ij}^{\Phi }$.

(4) $$V_{ij}^{\Phi }=V_{ij}\left\{\left(x^{I},\;y^{I}\right)\;|\;x^{I}=i,\;y^{I}\epsilon \;\left[1,\cdots ,W\right]\right\}\cup \;V_{ij}\left\{\left(x^{II},\;y^{II}\right)\;|\;x^{II}\epsilon \;\left[1,\cdots ,H\right],\;y^{II}=j\right\}\;\;\begin{array}{c} i\;\epsilon \;\left[1,\cdots ,H\right],\;j\;\epsilon \;\left[1,\cdots ,W\right] \end{array}$$

In this way, the residual part between the input *F* ∈ ${\mathbb{R}}^{H\times W\times C}$ and the output *F′* ∈ ${\mathbb{R}}^{H\times W\times C}$ can be obtained as follows:

(5) $$\begin{array}{c} F_{ij}^{\prime }=\sum V_{ij}^{\Phi }\;\cdot \;A_{ij}+F_{ij\;\;}\;\;i\epsilon \left[1,\;\cdots ,H\right],j\epsilon \left[1,\cdots ,W\right] \end{array}$$

where $F^{\prime }$ is the output of this module. In CCA part, the output $F^{\prime }$ is labelled as $F_{CCA}^{\prime }$. The implementation of the RCCA part will be introduced in Section 3.2.

### 3.2. Introduction of the RCCA Module

#### 3.2.1. Design and Realization of the RCCA Module

**Figure 5:**
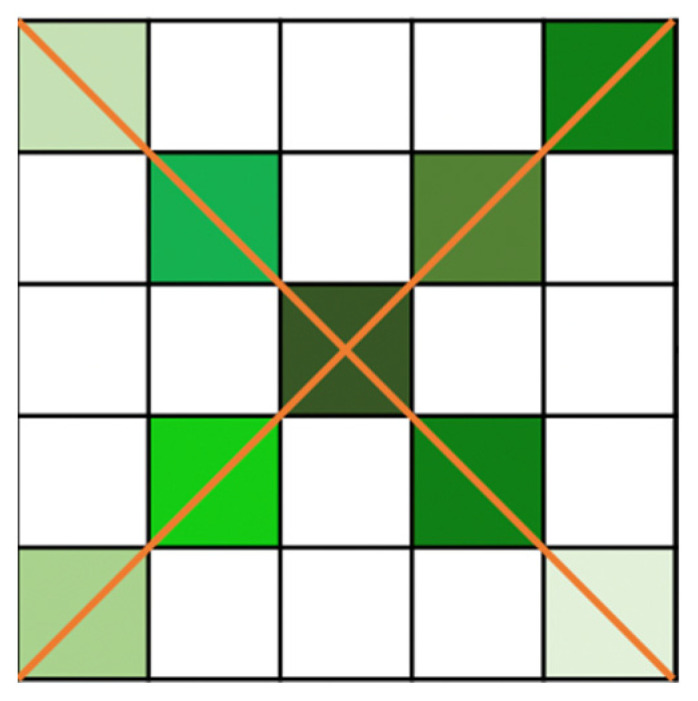
The attention area of RCCA module.

As mentioned in previous chapters, the RCCA module is a complement of the directionality in the attention area. As shown in Figure 5, the RCCA module can let the target pixel receive attention information from oblique directions.

**Figure 6:**
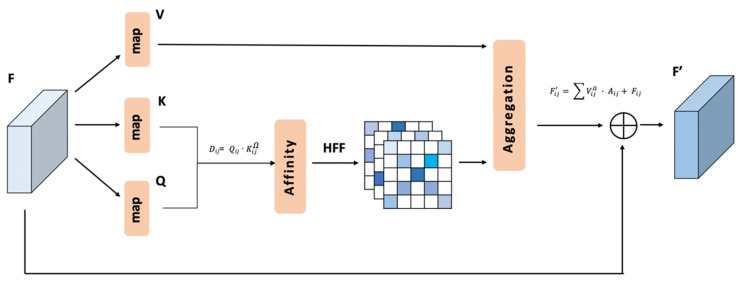
The structure of RCCA module.

The attention area needs to be extracted. For the pixels at different locations, the size of the attention area is different. The structure of RCCA module is shown in Figure 6.

First, we represent the dimension of *F* ∈ ${\mathbb{R}}^{(H\prime /8)\times (W\prime /8)\times \;C}$ as *F* ∈ ${\mathbb{R}}^{H\times W\times C}$. 

In the RCCA module part, the local feature map *F*∈ ${\mathbb{R}}^{H\times W\times C}$, the output of the backbone part, is mapped to *K* ∈ ${\mathbb{R}}^{H\times W\times C}\;$, query *Q* ∈ ${\mathbb{R}}^{H\times W\times C}$ and value *V* ∈ ${\mathbb{R}}^{H\times W\times C}$ (see Equation (2)).

For each position (i,j), in the spatial dimension of *Q*, we can obtain a vector *Q_ij_* ∈ ${\mathbb{R}}^{C}$. Then, for this position in feature map *K*, the channel vectors of the rotated crisscross area can be represented by $K_{ij}^{\Omega }$.

(6) $$K_{ij}^{\Omega }=K_{ij}\left\{\left(x^{I},\;y^{I}\right)\;|\;x^{I}=\alpha ,\;y^{I}=i+j-\alpha ,\;\;\alpha \;\epsilon \;A\left(i,j\right)\right\}\cup \;K_{ij}\left\{\left(x^{II},\;y^{II}\right)\;|\;x^{II}=\beta ,\;y^{II}=i-j+\beta ,\;\;\beta \;\epsilon \;Β\left(i,j\right)\right\}\;\;\begin{array}{c} i\;\epsilon \;\left[1,\cdots ,H\right],\;j\;\epsilon \;\left[1,\cdots ,W\right] \end{array}$$

$\left\{\left(x^{I},\;y^{I}\right)|\ldots \right\}$ and $\left\{\left(x^{II},\;y^{II}\right)|\ldots \right\}$ are two-point groups that represent two oblique lines in the rotated crisscross area. A(i,j) and Β(i,j) represent the initial position of the sampling area, which can be obtained as follows:

(7) $$\begin{array}{c} A\left(i,j\right)=\{\begin{array}{cc} \left\{0,\cdots ,i+j\right\} & \;\;if\;i+j\leq H\;\\ \left\{i+j-H,\cdots ,\;W\right\} & else \end{array} \end{array}$$

(8) $$\begin{array}{c} Β\left(i,j\right)=\{\begin{array}{cc} \left\{j-i,\cdots ,W\right\} & \;\;if\;i\leq j\;\\ \left\{0,\cdots ,\;j-i+H\right\} & \;else\; \end{array} \end{array}$$

For each position (i,j), we can apply an affinity operation to obtain the raw attention map *D* as follows:

(9) $$\begin{array}{c} \;D_{ij}=\;Q_{ij}\;\cdot \;K_{ij}^{\Omega }\;\;\;\;i\epsilon \left[1,\cdots ,H\right],j\epsilon \left[1,\cdots ,W\right] \end{array}$$

The $D_{ij}$ ∈ *D* represents the correlation between $Q_{ij}$ and $K_{\Omega ij}$.

Then, we use a heterogeneous filter function to process $D_{ij}$ as follows:

(10) $$\begin{array}{c} \;A_{ij}=HFF\left(\;D_{ij}\;\right) \end{array}$$

where $A_{ij}$ is the feature vector of attention map *A* at position (i,j).

At each position (i,j) in the spatial dimension of *V*, we can obtain the channel vectors of the rotated crisscross area, which can be represented by $V_{ij}^{\Omega }$:

(11) $$V_{ij}^{\Omega }=V_{ij}\left\{\left(x^{I},\;y^{I}\right)\;|\;x^{I}=\alpha ,\;y^{I}=i+j-\alpha ,\;\;\alpha \;\epsilon \;A\left(i,j\right)\right\}\cup \;V_{ij}\left\{\left(x^{II},\;y^{II}\right)\;|\;x^{II}=\beta ,\;y^{II}=i-j+\beta ,\;\;\beta \;\epsilon \;Β\left(i,j\right)\right\}\;\;\;\;\begin{array}{c} \;i\epsilon \left[1,\;\cdots ,H\right],j\epsilon \left[1,\cdots ,W\right] \end{array}$$

For each position (i,j), an aggregation process is used to process the channel vector $A_{ij}$ and the channel vectors of rotated crisscross attention area $V_{ij}^{\Omega }$ to obtain the residual part between the input *F* ∈ ${\mathbb{R}}^{H\times W\times C}$ and the output *F’* ∈ ${\mathbb{R}}^{H\times W\times C}$ as follows:

(12) $$\begin{array}{c} \;F_{ij}^{\prime }=\sum V_{ij}^{\Omega }\;\cdot \;A_{ij}+F_{ij\;\;}\;i\epsilon \left[1,\;\cdots ,H\right],j\epsilon \left[1,\cdots ,W\right] \end{array}$$

$F^{\prime }$ is the output of this module, which is generated by the combination of $F_{ij}^{\prime }$. In RCCA part, the output $F^{\prime }$ is labelled as $F_{RCCA}^{\prime }$.

The pseudocode of the RCCA module is attached in Algorithm 1:
**Algorithm 1.** The process of RCCA attention.
Input: feature map F Output: attention feature map F′ Initialize: K, Q,V=F **1**:    for i in  {1,⋯, H}  do **2**:   for j in  {1,⋯, W} do /* According to the points groups ($x^{I},y^{I}$) and ($x^{II},y^{II})$, get feature value of the RCCA region positions in K. */ /* A(i,j) and Β(i,j) represent the range of index on the horizontal axis of the feature map. */ **3**: $\;\;\;\begin{array}{c} A\left(i,j\right)=\{\begin{array}{cc} \left\{0,\cdots ,i+j\right\} & \;\;if\;i+j\leq H\;\\ \left\{i+j-H,\cdots ,\;W\right\} & else \end{array} \end{array}$ **4**: $\;\;\;\begin{array}{c} Β\left(i,j\right)=\{\begin{array}{cc} \left\{j-i,\cdots ,W\right\} & \;\;if\;i\leq j\;\\ \left\{0,\cdots ,\;j-i+H\right\} & \;else\; \end{array} \end{array}$ **5**:    for α in A(i,j) do **6**: $\;\;\;\;\;\;x^{I}\leftarrow \alpha$ **7**: $\;\;\;\;\;y^{I}\leftarrow \;\left(i+j\right)-\alpha$ **8**:    for β in Β(i,j) do **9**: $\;\;\;\;\;x^{II}\leftarrow \beta$ **10**: $\;\;\;\;\;y^{II}\leftarrow \;\left(i-j\right)+\beta$ /* $K_{ij}^{\Omega }$ represents the channel vectors sets of the RCCA region in K. */ **11**: $\;\;\;K_{ij}^{\Omega }\;\epsilon \;K_{ij}\left(x^{I},y^{I}\right)\cup K_{ij}\left(x^{II},y^{II}\right)$ /* Search Q corresponding to the position in K and then get the energy map E */ **12**: $\;\;\;\;E_{ij}=Q_{ij}\;\cdot \;K_{\Omega }\;$ **13**:     end **14**:   end /* Apply activation function HFF to the energy map, HFF is defined in 3.3 */ **15**:    E←HFF(E) **16**:    for  i in  {1,⋯, H} do **17**:   for j in  {1,⋯, W} do /* According to the point set ($x^{I},y^{I}$) and ($x^{II},y^{II})$, get feature value of RCCA region positions in V */ **18**: $\;\;\;V_{ij}^{\Omega }\;\epsilon \;V_{ij}\left(x^{I},y^{I}\right)\cup V_{ij}\left(x^{II},y^{II}\right)$ /* Aggregate E and V and $F_{ij}$ represents the residual structure */ **19**: $\;\;\;F_{ij}^{\prime }\leftarrow \;V_{\Omega }\;\cdot \;E_{ij}+F_{ij}$ **20**:     end **21**:   end

#### 3.2.2. Functional Merits of the RCCA Module

**Figure 7:**
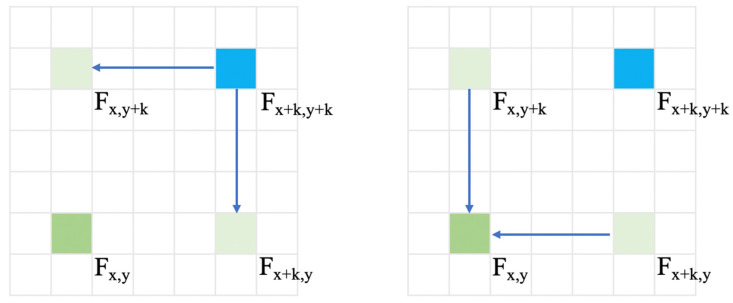
The recurrent mechanism.

When using the recurrent mechanism to relate position (x+k,y+k) to position (x,y), the process is shown in Figure 7.

In the first attention process, the value change of each position in the recurrent mechanism can be shown:

(13) $$\begin{array}{cc} F_{x+k,y}\left(CCA\right)= & Q_{x+k,y}\;\cdot K_{x+k,y}\left(x+k,y+k\right)\cdot F_{x+k,y}\left(x+k,y+k\right)+Q_{x+k,y}\\ & \cdot (\;K_{x+k,y}\{\left(x^{I},\;y^{I}\right)\;|\;x^{I}=x+k,\;y^{I}\epsilon \;\left[1,\cdots ,W\right],\;y^{I}\neq y+k\}\\ & \cup \;K_{x+k,y}\left\{\left(x^{II},\;y^{II}\right)\;|\;x^{II}\epsilon \;\left[1,\cdots ,H\right],\;y^{II}=y\right\})\\ & \cdot (F_{x+k,y}\left\{\left(x^{I},\;y^{I}\right)\;|\;x^{I}=x+k,\;y^{I}\epsilon \;\left[1,\cdots ,W\right],\;y^{I}\neq y+k\right\}\\ & \cup \;F_{x+k,y}\left\{\left(x^{II},\;y^{II}\right)\;|\;x^{II}\epsilon \;\left[1,\cdots ,H\right],\;y^{II}=y\right\}) \end{array}\;\begin{array}{c} =Q_{x+k,y}\;\cdot K_{x+k,y}\left(x+k,y+k\right)\cdot V_{x+k,y}\left(x+k,y+k\right)+\delta_{1} \end{array}$$

(14) $$\begin{array}{cc} F_{x,y+k}\left(CCA\right)= & Q_{x,y+k}\;\cdot K_{x,y+k}\left(x+k,y+k\right)\cdot F_{x,y+k}\left(x+k,y+k\right)+Q_{x,y+k}\\ & \cdot (K_{x,y+k}\{\left(x^{I},\;y^{I}\right)\;|\;x^{I}=x,\;y^{I}\epsilon \;\left[1,\cdots ,W\right]\}\\ & \cup \;K_{x,y+k}\left\{\left(x^{II},\;y^{II}\right)\;|\;x^{II}\epsilon \;\left[1,\cdots ,H\right],\;y^{II}=y+k,\;x^{II}\neq x+k\right\})\\ & \cdot (F_{x,y+k}\left\{\left(x^{I},\;y^{I}\right)\;|\;x^{I}=x,\;y^{I}\epsilon \;\left[1,\cdots ,W\right]\right\}\\ & \cup \;F_{x,y+k}\left\{\left(x^{II},\;y^{II}\right)\;|\;x^{II}\epsilon \;\left[1,\cdots ,H\right],\;y^{II}=y+k,\;x^{II}\neq x+k\right\}) \end{array}\;\begin{array}{c} =Q_{x,y+k}\;\cdot K_{x,y+k}\left(x+k,y+k\right)\cdot V_{x,y+k}\left(x+k,y+k\right)+\delta_{2} \end{array}$$

$Q_{x+k,y}\;\cdot K_{x+k,y}\left(x+k,y+k\right)$ is the similarity parameter of positions (x+k,y+k) and (x+k,y) and $Q_{x,y+k}\;\cdot K_{x,y+k}\left(x+k,y+k\right)$ is the similarity parameter of positions (x+k,y+k) and (x,y+k). $\delta_{1}$ and $\delta_{2}$ are the redundancy information which is caused by feature vectors of other positions. 

In the second attention process, the value change of each position in the recurrent mechanism can be shown:

(15) $$F_{x,y}\left(CCA\right)=\;Q_{x,y}\;\cdot K_{x,y}\left(x+k,y\right)\cdot F_{x,y}\left(x+k,y\right)+Q_{x,y}\;\cdot K_{x,y}\left(x,y+k\right)\cdot F_{x,y}\left(x,y+k\right)+\;\;\;Q_{x,y}\cdot (\;K_{x,y}\left\{\left(x^{I},\;y^{I}\right)\;|\;x^{I}=x,\;y^{I}\epsilon \;\left[1,\cdots ,W\right],\;y^{I}\neq y+k\right\}\cup \;\;\;K_{x,y}\left\{\left(x^{II},\;y^{II}\right)\;|\;x^{II}\epsilon \;\left[1,\cdots ,H\right],\;y^{II}=y+k,\;x^{II}\neq x+k\right\}\;\;)\cdot \;\;\;\;(\;F_{x,y}\left\{\left(x^{I},\;y^{I}\right)\;|\;x^{I}=x,\;y^{I}\epsilon \;\left[1,\cdots ,W\right],\;y^{I}\neq y+k\right\}\cup \;\;\;\;F_{x,y}\left\{\left(x^{II},\;y^{II}\right)\;|\;x^{II}\epsilon \;\left[1,\cdots ,H\right],\;y^{II}=y+k,\;x^{II}\neq x+k\right\}\;=\;Q_{x,y}\;\cdot K_{x,y}\left(x+k,y\right)\cdot F_{x,y}\left(x+k,y\right)+Q_{x,y}\;\cdot K_{x,y}\left(x,y+k\right)\cdot F_{x,y}\left(x,y+k\right)+\;\delta_{3}$$

$Q_{x,y}\;\cdot K_{x,y}\left(x+k,y\right)$ is the similarity parameter of positions (x+k,y) and (x,y) and $Q_{x,y}\;\cdot K_{x,y}\left(x,y+k\right)$ is the similarity parameter of positions (x,y+k) and (x,y). $\delta_{3}$ is the redundancy information in this step.

Therefore, when we generate two processes together, we can obtain $f_{x,y}\left(CCA\right)$ as follows:

(16) $$\;F_{x,y}\left(CCA\right)=Q_{x,y}\;\cdot K_{x,y}\left(x+k,y\right)\times \;\left(Q_{x+k,y}\;\cdot K_{x+k,y}\left(x+k,y+k\right)\times F_{x+k,y+k}\left(CCA\right)+\delta_{1}\right)+\;\;\;\;\;\;\;Q_{x,y}\;\cdot K_{x,y}\left(x,y+k\right)\times \;\left(Q_{x,y+k}\;\cdot K_{x,y+k}\left(x+k,y+k\right)\times F_{x+k,y+k}\left(CCA\right)+\delta_{2}\right)+\delta_{3}$$

After summing all the redundancy information to ∑δ, we can obtain the following:

(17) $$\begin{array}{cc} F_{x,y}\left(CCA\right)= & Q_{x,y}\;\cdot K_{x,y}\left(x+k,y\right)\times Q_{x+k,y}\;\cdot K_{x+k,y}\left(x+k,y+k\right)F_{x+k,y+k}\left(CCA\right)+\\ & Q_{x,y}\;\cdot K_{x,y}\left(x,y+k\right)Q_{x,y+k}\;\cdot K_{x,y+k}\left(x+k,y+k\right)F_{x+k,y+k}\left(CCA\right)+\sum \delta \end{array}$$

**Figure 8:**
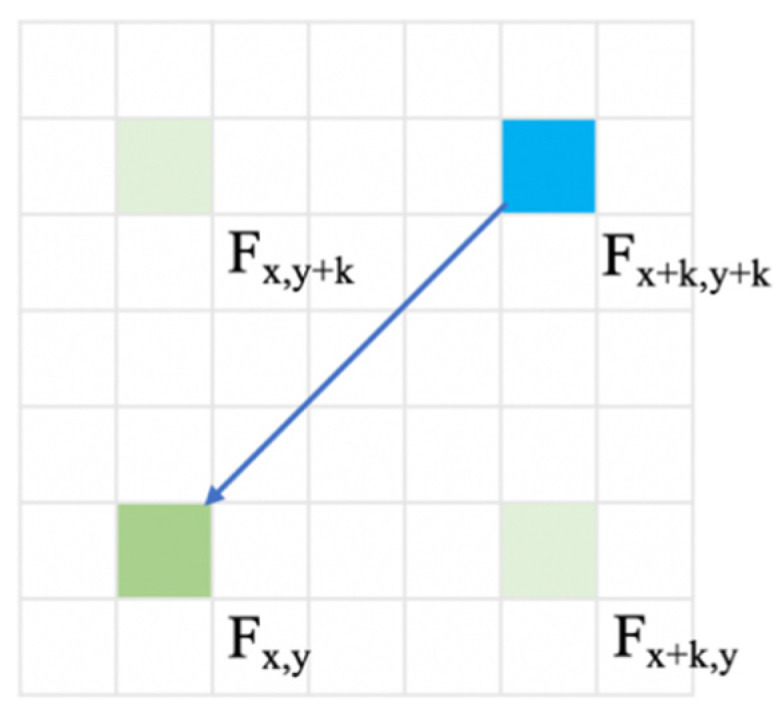
The relation information transference in RCCA.

When using the RCCA module, the relation between (x+k,y+k) and (x,y) is shown in Figure 8:

The value change can be represented as follows:

(18) $$\begin{array}{cc} F_{x,y}\left(RCCA\right)= & Q_{x,y}\;\cdot K_{x,y}\left(x+k,y+k\right)\cdot F_{x,y}\left(x+k,y+k\right)+\\ & \;Q_{x,y}\;\cdot (\;K_{x,y}\left\{\left(x^{I},\;y^{I}\right)\;|\;x^{I}=\alpha ,\;y^{I}=x+y-\;\alpha ,\;\;\alpha \;\epsilon \;Α\left(x,y\right)\right\}\cup \\ & \;\;K_{x,y}\left\{\left(x^{II},\;y^{II}\right)\;|\;x^{II}=\beta ,\;y^{II}=x-y+\;\beta ,\;\;\beta \;\epsilon \;Β\left(x,y\right),\beta \neq x+k\;\right\}\;)\cdot \\ & \;(\;F_{x,y}\left\{\left(x^{I},\;y^{I}\right)\;|\;x^{I}=\alpha ,\;y^{I}=x+y-\;\alpha ,\;\;\alpha \;\epsilon \;Α\left(x,y\right)\right\}\cup \\ & \;F_{x,y}\left\{\left(x^{II},\;y^{II}\right)\;|\;x^{II}=\beta ,\;y^{II}=x-y+\;\beta ,\;\;\beta \;\epsilon \;Β\left(x,y\right),\beta \neq x+k\;\right\}\;)\\ = & \;Q_{x,y}\;\cdot K_{x,y}\left(x+k,y+k\right)\cdot F_{x+k,y+k}\left(RCCA\right)+\;\delta_{4} \end{array}$$

$Q_{x,y}\;\cdot K_{x,y}\left(x+k,y+k\right)$ is the similarity parameter of positions (x+k,y+k) and (x,y). $\delta_{4}$ is the information redundancy caused by feature vectors of other positions.

When we compare the similarity parameters of $F_{x,y}\left(CCA\right)$ and $F_{x,y}\left(RCCA\right)$, the ratio r can be represented as follows:

(19) $$\begin{array}{c} r=\frac{Q_{x,y}\;\cdot K_{x,y}\left(x+k,y\right)\times Q_{x+k,y}\;\cdot K_{x+k,y}\left(x+k,y+k\right)+Q_{x,y}\;\cdot K_{x,y}\left(x,y+k\right)Q_{x,y+k}\;\cdot K_{x,y+k}\left(x+k,y+k\right)}{Q_{x,y}\;\cdot K_{x,y}\left(x+k,y+k\right)} \end{array}$$

When positions (x+k,y+k) and (x,y) are homogeneous and (x+k,y) and (x,y+k) are heterogeneous compared to (x,y), the similarity parameters of (x+k,y+k) and (x,y) will be extremely low in the recurrent mechanism. When using the RCCA module under the same conditions, which can let these two positions be related directly, the similarity parameter can be high, corresponding to the real situation. The ratio r can sometimes be very low because the recurrent mechanism will cause similarity distortion.

**Figure 9:**
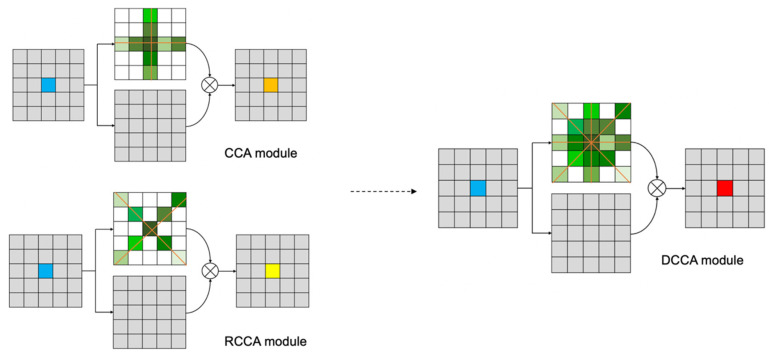
The relation information transference in RCCA.

DCCA module consists of CCA module and RCCA module. It can also distribute the weight of each attention module according to the statistics of the energy map in the SAF module. The shape of the attention area allows each pixel to receive contextual information from eight directions, as shown in Figure 9.

**Figure 10:**
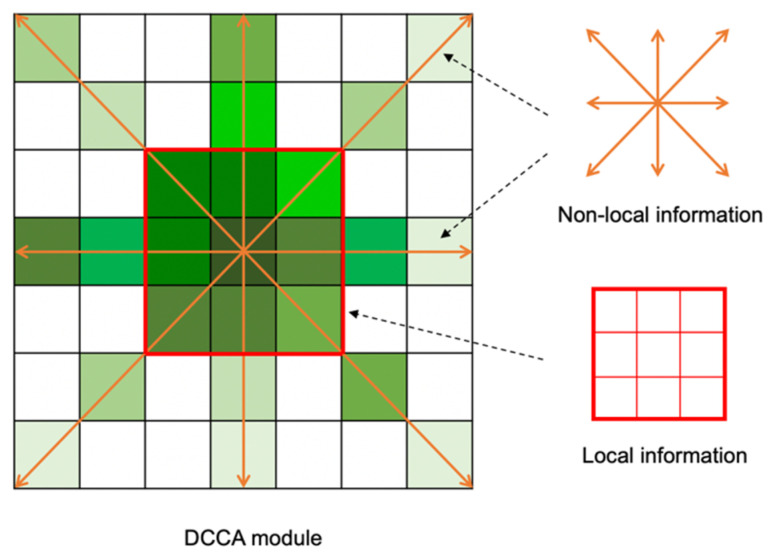
The local and non-local information in DCCA module.

Considering the local information extracted by 3×3  convolution, the DCCA module can let each pixel perceive the local information and eight-direction nonlocal information, as shown in Figure 10.

#### 3.2.3. Computational Advantages of the RCCA Module

From the aspect of the theory of the algorithm, we will analyze the computational cost based on the attention information received by each pixel. In CCNet, each pixel receives the information from the pixels in the same column and row. The total computational cost can be described as follows:

(20) $$O\left(CCA\right)=\iint_{D}^{a}f\left(i,j\right)didj\;\;\;\;\;\;\;\;\;\;D=\{(i,\;j)\;|\;i\;\epsilon \;\left[1,\cdots ,H\right],\;j\;\epsilon \;\left[1,\cdots ,W\right]\}\;f\left(i,j\right)=H+W-1$$

So, we can conclude that:

*O*(*CCA*) = *H* × *W* × (*H* + *W* − 1) (21)

The computational cost of RCCA module can be calculated as follows:

(22) $$O\left(RCCA\right)=\iint_{D}^{a}f\left(i,j\right)didj\;\;\;\;\;\;\;\;\;\;\;D=\{(i,\;j)\;|\;i\;\epsilon \;\left[1,\cdots ,H\right],\;j\;\epsilon \;\left[1,\cdots ,W\right]\}\;\;\;\;\;\;\;\;\;\;\;\begin{array}{c} A\left(i,j\right)=\{\begin{array}{cc} \left\{0,\cdots ,i+j\right\} & \;\;if\;i+j\leq H\;\\ \left\{i+j-H,\cdots ,\;W\right\} & else \end{array} \end{array}\;\;\;\;\;\;\;\;\;\begin{array}{c} Β\left(i,j\right)=\{\begin{array}{cc} \left\{j-i,\cdots ,W\right\} & \;\;if\;i\leq j\;\\ \left\{0,\cdots ,\;j-i+H\right\} & \;else\; \end{array} \end{array}\;f\left(i,j\right)=card\left(A\cup B\right)$$

The function card( ) represents the number of elements in the set. So, we can conclude that:

(23) $$O\left(RCCA\right)=H\times W\times \left(H+W-1\right)\times \frac{2}{3}=\frac{2}{3}\;O\left(CCA\right)\;O\left(DCCA\right)=O\left(CCA\right)+O\left(RCCA\right)=\frac{5}{3}\times H\times W\times \left(H+W-1\right)$$

That is to say, theoretically, the computational cost of the RCCA module is two-thirds of that of a single CCA. The computational cost of the DCCA module is five-thirds of that of a single CCA. The computational cost of Non-local Network [2] can be described as:

(24) O(Non local)=H×W×(H×W)≫O(DCCA)

Through comparison, it can be concluded that the computational cost of DCCA module is much smaller than that of Non-local Network.

### 3.3. Heterogeneous Filter Function and Output Part

#### 3.3.1. Heterogeneous Filter Function

In energy processing, there is an unevenly distributed sequence of energy values. These energy groups come from the values taken in different attention areas. In the DCCA module, we need to process these energy values to assign weights. The value of energy represents the similarity between point pairs. In such a process, we expect that this filter function can benefit our classification problem. That is, the filter function needs to remove the influence of the heterogeneous part, which can let each part receive more relation information from the homogeneous part.

**Figure 11:**
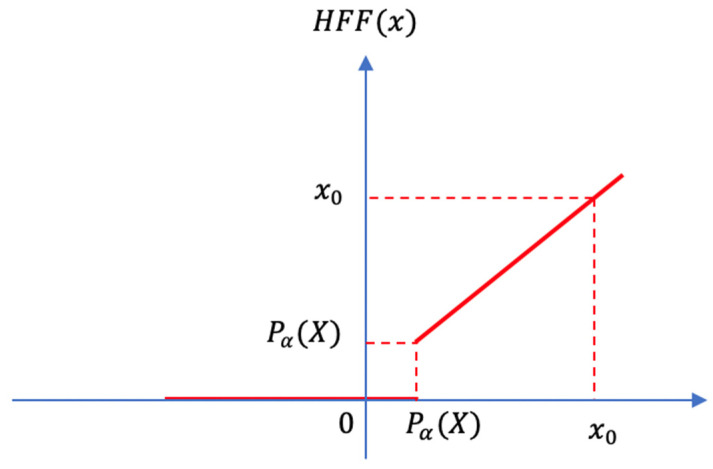
HFF.

We design the Heterogeneous Filter Function (HFF) to help us remove the influence of a part of the energy values that are relatively low in one energy group. The implementation details are shown in Figure 11:

The mathematical formula of HFF can be expressed as follows:

(25) $$\begin{array}{c} HFF\left(x\right)=\{\begin{array}{c} x\;\;\;\;\;if\;x\geq P_{\alpha }\left(X\right)\\ 0\;\;\;\;\;\;\;else\;\;\;\;\;\;\;\;\;\;\;\;\;\; \end{array} \end{array}$$

**Figure 12:**
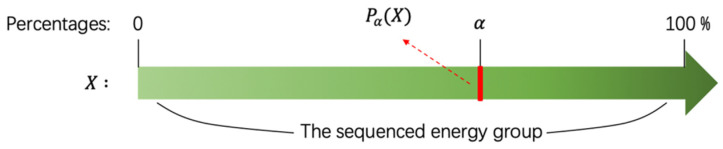
The position function $P_{\alpha }\left(X\right)$.

We introduce a position parameter α to determine how large the lost part is. We arrange the value of energy from small to large in a sequence. The parameter α is a percentage. The position function $P_{\alpha }\left(X\right)$ refers to the value at the α position in sorting from small to large, as shown in Figure 12.

**Figure 13:**
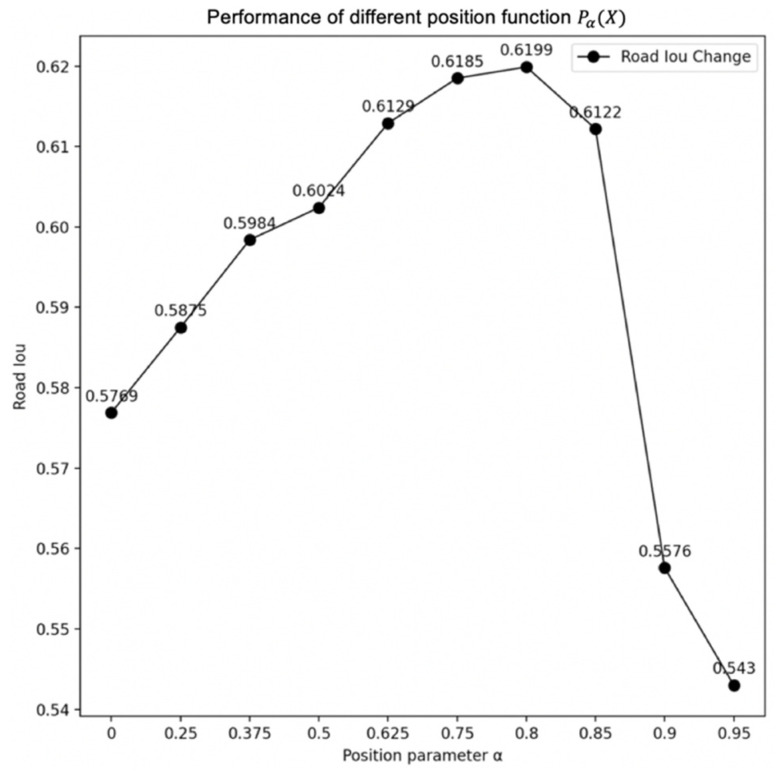
The performance of different position function $P_{\alpha }\left(X\right)$.

The energy value, which is smaller than $P_{\alpha }\left(X\right)$, will be fixed to 0. Consequently, the lost part of the energy will have no influence in the attention process. The existence of the position function $P_{\alpha }\left(X\right)$ ensures that the DCCA model still has the ability to identify homogeneous and heterogeneous regions for the change of energy distribution. In the experiment, it can be found that the most proper position parameter α is often related to the road pixel percentages of the images in the training dataset, as shown in Figure 13.

In Figure 13 we choose the road intersection over union (IOU) as a statistical index to show the performance of each different α. When the position parameter α is approximately 0.8, corresponding to the average percentage of nonroad parts in images, the performance will be the best. Therefore, α needs to be matched with the percentage of non-road parts in the dataset.

**Figure 14:**
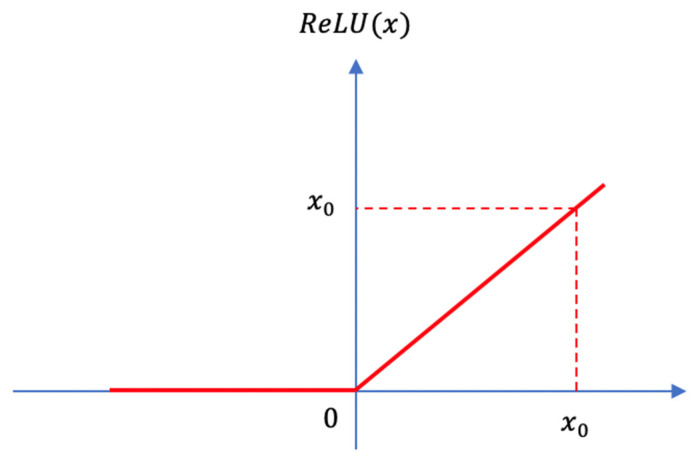
ReLU function.

For such a type of data processing problem, the ReLU function is often used in traditional methods, which is shown in Figure 14.

The function can be expressed in the following form:

(26) $$\begin{array}{c} ReLU\left(x\right)=\{\begin{array}{c} x\;\;\;\;\;\;if\;x\geq 0\\ 0\;\;\;\;\;\;\;\;\;\;else\;\;\;\; \end{array}\; \end{array}$$

In the traditional ReLU function, there is an absolute threshold for numerical filtering. In most cases, this threshold is 0, similar to the example in Figure 14. However, in the road extraction problem, the threshold for judging the energy cannot be a fixed number. In different samples, the value of energy will vary greatly; therefore, we need to perform the filter process according to the energy value ranking in the sampling group, which is the reason why the ReLU function is not suitable for use in the DCCA module.

#### 3.3.2. Output Part: SAF Module

In the output part, the SAF module can recognize the road shape through the mean values of energy in two different attention intervals. The mean value of the energy reflects the homogeneity and heterogeneity in the sampling area, which will be illustrated in the experimental section.

SAF module consists of these steps:

For each position u, we take the mean value of the energy vector $A_{u}^{CCA}$ ∈ ℝ^H+W−1^ and $A_{u}^{RCCA}$∈ ℝ^L^ (which has been introduced in Section 3.2) of the crisscross and rotated crisscross attention modules: $E_{u}^{CCA}$ and $E_{u}^{RCCA}$.

The softmax function is used to process the two mean values and obtain the weights of the two attention modules, $\omega_{CCA}$ and $\omega_{RCCA}$. This can be shown as follows:

(27) $$\begin{array}{c} \left(\;\omega_{CCA},\;\omega_{RCCA}\;\right)=SoftMax\left(E_{u}^{CCA},\;E_{u}^{RCCA}\;\right) \end{array}$$

The weights of the two attention modules represent the prediction of road shape from the SAF module, which is the key part of the self-adaptive mechanism. If one attention module obtains a higher weight in one point, the road shape around this point is more likely to be the shape of this attention module. Under this condition, in the SAF module, a higher weight can let this point receive more relation information from the road area.

Multiply the weights by the outputs of the two attention modules to obtain the vector of fusion result in position u.

(28) $$\begin{array}{c} F_{u}^{output\;\prime }=\omega_{CCA}\times F_{u}^{CCA\;\prime }+\omega_{RCCA}\times F_{u}^{RCCA\;\prime } \end{array}$$

**Figure 15:**
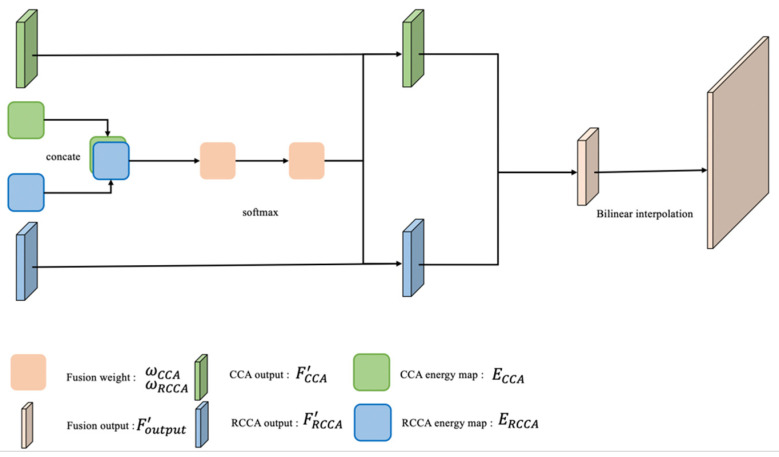
The structure of the output part.

$F_{u}^{output\;\prime }$ is combined in the spatial dimension to obtain $F_{output}^{\prime }$, the fusion result. The function g represents the upsampling process. The structure of the output part is shown in the Figure 15.

(29) $$\begin{array}{c} F_{output}^{\prime }=g\left(F_{output}^{\prime }\right) \end{array}$$

## 4. Experiment

### 4.1. Dataset and Implement Details

The dataset of this experiment is based on the DeepGlobe Road Extraction Challenge [43]. The size of the road image has been changed from 1024 pixels to 256 pixels by downsampling. To emphasize the function of our special attention mechanism, we select those images that have distinctive direction features. The road directions on these images are evenly distributed in space. Then, we create two subdatasets, which are both parts of the entire dataset. The first subdataset consists of all the images that mainly include horizontal and vertical roads, whose angles between their direction and the horizontal or vertical direction are less than 22.5°.

**Figure 16:**
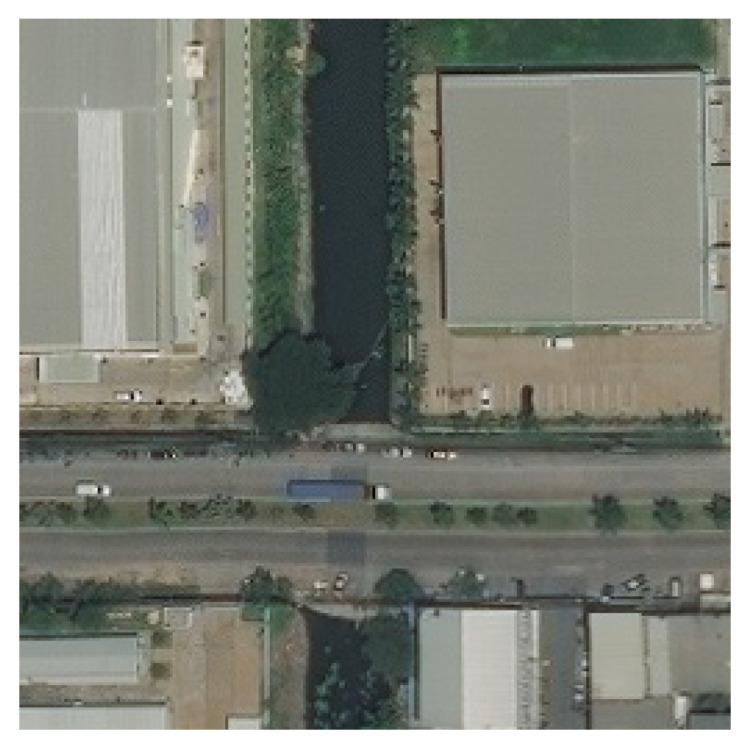
The image in CC dataset.

This subdataset is called the crisscross dataset (CC dataset). In Figure 16, an example image is shown, which contains two horizontal roads.

**Figure 17:**
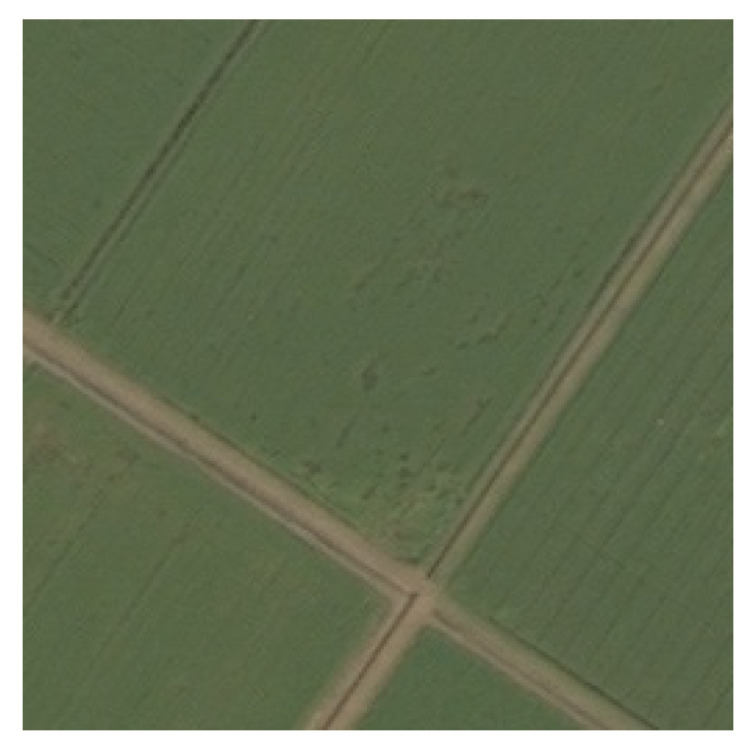
The image in RCC dataset.

Another subdataset is the residual part in the total dataset, which contains images with slope roads inside, called the rotated crisscross dataset (RCC dataset). In Figure 17, an example image is shown, and it contains two slope roads.

We implement different attention methods and compare the results to analyse the advantages of our special attention method to a great extent. The difference between the CCA module and RCCA module is the attention area. For each pixel in one feature map, we will generate two energy maps for two different attention areas. In these energy maps, we will analyse the energy distribution to determine whether the energy can reflect the homogeneity and heterogeneity of pixels. Then, we use the SAF module to distribute the weight of the CCA module and RCCA module according to the energy map. In this way, we can obtain the result of the DCCA module and compare it with the results of the CCA module and RCCA module. In detail, we will also select some typical examples to illustrate the merits of our DCCA module.

### 4.2. Results of Different Attention Methods

In this part, we select the IOU as the statistical index. In this two-classification problem, we set the classification labels as road and background.

The road IOU and mean IOU are indices that we choose to compare the results of different attention modules. Mean IOU is the mean value of the Road IOU and the Background IOU.

When applying the CCA module to the complete dataset and two subdatasets, the CCA module shows the highest result when being used in the crisscross dataset from the perspectives of both mean IOU and road IOU in Table 1. The road IOU and mean IOU of the complete dataset are at the mean level of the results of the CC dataset and RCC dataset. From these two characteristics, we can conclude that the CCA module has a better processing effect on crisscross-shaped roads. It seems powerless for the road in the rotated crisscross shape. The reason for its relatively poor performance on the RCC dataset is that the CCA module cannot effectively extract the geometric information of rotated crisscross-shaped roads.

Table 2 shows that the road IOU and mean IOU of the CC dataset are still the highest. Although these 2 indicators of the RCC dataset are still not as good as those of the CC dataset, there is still a rise compared to the result of the CCA module on the RCC dataset. This result shows that the RCCA module can extract the geometric information of the rotated crisscross shape better than the CCA module.

When applying the DCCA module to three different datasets in Table 3, the road IOU and mean IOU are the best. The performance of the CCA module, RCCA module and DCCA module on the complete dataset can show the properties of each attention method. The results of three different attention modules on the complete dataset can reveal the high performance of the DCCA module.

**Figure 18:**
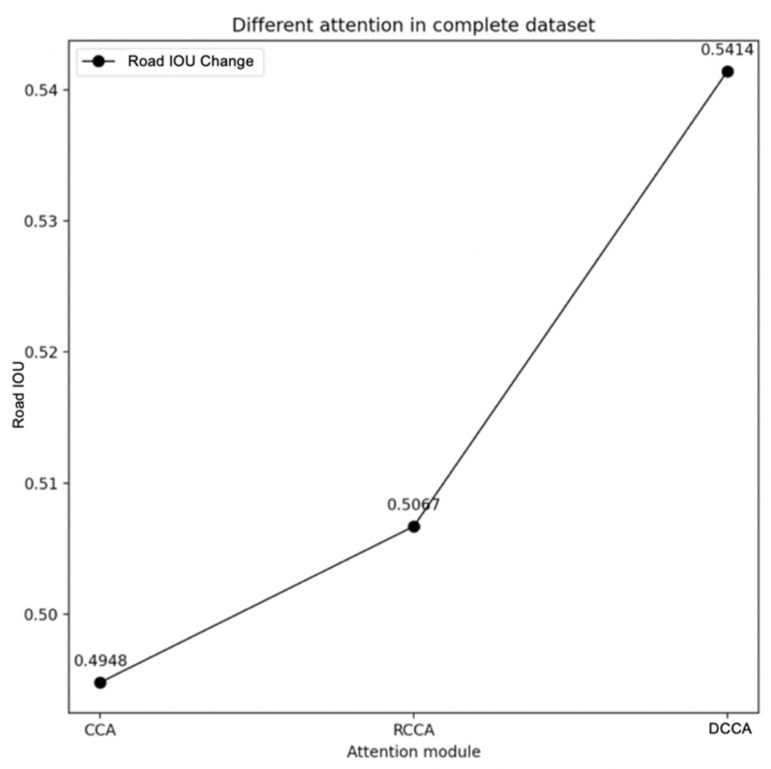
The result of different attention on complete dataset.

In Table 4, the performance of the DCCA module on the complete dataset is significantly ahead of that of the CCA module and RCCA module. Focusing on the road IOU, the advantages of the DCCA module are shown in Figure 18 as follows:

In Figure 18, from the perspective of the road IOU indicator, the result of the DCCA module generally leads the CCA and RCCA by 4 percent.

In order to illustrate the performance of the DCCA module, we also incorporate a 30° module into the DCCA module for experimentation. At the same time, we also tested the performance of Non-local Network [2], CBAM [9] and PSPNet [17] mentioned above. The final results are shown in the Table 5.

We conclude that adding more directional modules and letting each pixel perceive the information of all pixels (Non-local) will improve the Mean IOU of the road and the background. But the Road IOU will not necessarily improve. 

From the above comparison, we can also conclude that the DCCA module is in the leading position on the Road IOU. Only the Non-local method is similar to the result of the DCCA module. The computational cost we got in Section 3.2.3 shows that the computational cost of Non-local is much greater than that of the DCCA module. The performance of DCCA module is significantly better than CBAM and PSPNet. This reflects the advantages of the DCCA module in road extraction problems.

### 4.3. Explanation of the Result

The raw energy, $D_{ij}$ iϵ[1,⋯,H],jϵ[1,⋯,W], is calculated by the inner product of the 2-pixel channel vector, which reflects the similarity of the centre pixel and the pixels in the sampling area. In the road extraction, since the similarity between the road points is higher than the similarity between the road and other features, the energy calculated between the points on the road will be very high in this way.

Based on this principle, the calculation of energy helps us distinguish roads from nonroads. In the DCCA module, for one point, two attention areas will be considered, the crisscross area and rotated crisscross area, which regard this point as the centre point. The energy inside these two areas is calculated, and then the weight distribution is performed according to the calculated energy in these two areas.

The position of energy calculation processing is in front of the attention process; therefore, whether the energy can correctly reflect the similarity between the two points becomes the key point. The following will take several typical samples to analyse the numerical characteristics of energy in the similarity between road points.

#### 4.3.1. Example with Only Crisscross Shaped Roads

**Figure 19:**
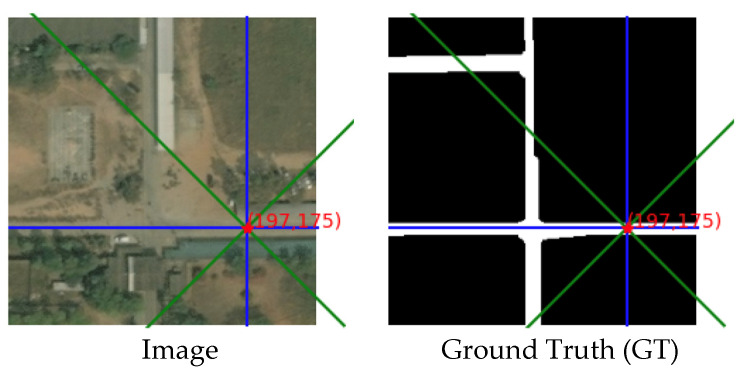
The image and its corresponding ground truth.

In Figure 19, there is an image in the dataset and its corresponding ground truth. The selected red points on the image are used to analyse the energy. To generate the energy map, we calculate the inner product of this pixel and each pixel in the image. There are two sampling areas in the energy map: crisscross area and rotated crisscross area. The crisscross area is represented by the blue lines, while the rotated crisscross area is represented by the green lines.

**Figure 20:**
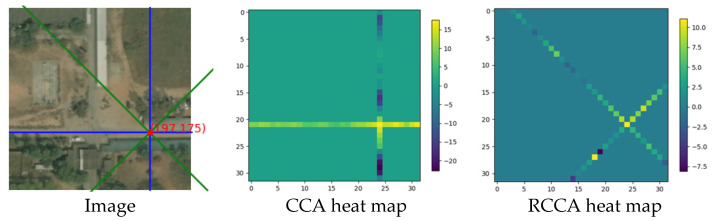
The heat map of energy distribution.

(1) The energy heat map:

The energy heat map for these two areas is shown as follows:

In Figure 20, the heat map of the energy distribution of two different sampling areas is shown.

The fixed point is in the road; therefore, most of the points that are homogeneous with the fixed point are distributed on the road. From the energy map, we can see clearly that when the sampling area comes to the road area, the energy value becomes higher than that of the non-road area. This rule can be seen from the brightness of the pixels in the image.

**Figure 21:**
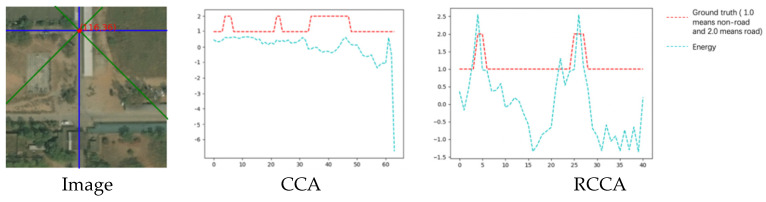
The energy line chart of two sampling area.

(2) The energy line chart:

The energy value of each sampled pixel can be displayed more clearly through the curve. In Figure 21, the abscissa represents the pixel points in two-point groups of the CCA module: $\left\{\left(x^{I},\;y^{I}\right)|\ldots \right\}$ and $\left\{\left(x^{II},\;y^{II}\right)|\ldots \right\}$. For the red line, the ordinate is used to distinguish roads from non-roads, which is explained in the legend. The ordinate is the value of energy for the blue line. The abscissa represents the pixel label, and each pixel in the sampling interval has a label number. It can be seen from these two figures that when energy is used to express the similarity of two homogeneous points, the value will become larger compared to the case of heterogeneous points. The distribution of the entire energy value is highly similar to the distribution of the ground truth.

(3) The energy table:

In the two sampling areas, the mean value of energy for road points and non-road points can be displayed in Table 6:

The fixed point is on the road. The energy between this point and other road points is higher than the energy between this point and other non-road points. It can be concluded that the energy map is very distinguishable for the homogeneity and heterogeneity of points. 

Such an energy map can help the attention process play an important role in determining to what extent the point pair needs to be focused.

**Figure 22:**
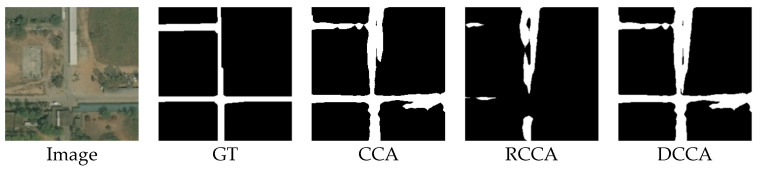
Image, ground truth and the output compilation.

(4) The output collection:

The outputs of this image are shown below:

Figure 22 shows that the result of the CCA module is better than the result of the RCCA module. The CCA module can help to focus on more areas of roads because roads are crisscross-shaped. Due to the distinguishability of the energy map, the CCA module can help each pixel pay more attention to the road area. Because of the SAF module inside the DCCA module, the result of the DCCA module remains the advantage of the CCA module.

#### 4.3.2. Example with Only Rotated Crisscross Shaped Roads

**Figure 23:**
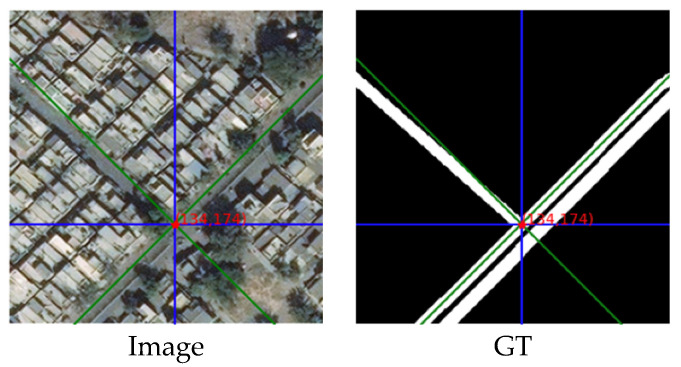
The image and its ground truth.

The next example is for the road in a rotated crisscross shape:

Figure 23 shows an image with a rotated crisscross-shaped road and its corresponding ground truth. The selected red points on the image are used to analyse the energy.

**Figure 24:**
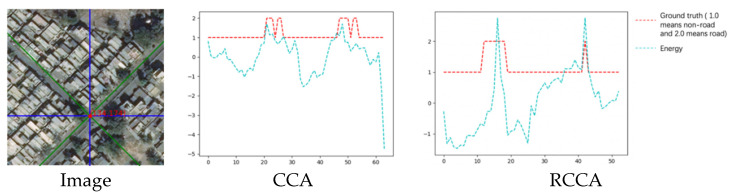
The energy line chart of two sampling area.

(1) The energy line chart:

By the same way to calculate the energy, the energy value of each sampled pixel can be displayed clearly through the curve:

In Figure 24, the abscissa also represents the pixel points in two-point groups of the RCCA module: $\left\{\left(x^{I},\;y^{I}\right)|\ldots \right\}$ and $\left\{\left(x^{II},\;y^{II}\right)|\ldots \right\}$. In this rotated crisscross-shaped road example, the rotated crisscross-shaped sampling area contains more road pixels. We can obtain the same conclusion as the last example that the distribution of the entire energy value is highly similar to the distribution of the ground truth.

(2) The energy table:

In the two sampling areas, the mean value of energy for road points and non-road points can be displayed in Table 7:

The distinguishability of the energy map is still clear in this rotated crisscross-shaped road sample.

**Figure 25:**
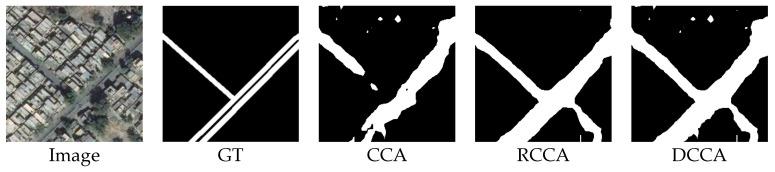
The image, ground truth and the output compilation.

(3) The output collection:

The output of this sample by the CCA module and RCCA module is shown in Figure 25:

Figure 25 shows that the result of the RCCA module is better than that of the CCA module. The RCCA module truly predicts more road parts, which means that in this case, the RCCA module can focus on more road areas. In this rotated crisscross-shaped road image, the DCCA module can distribute a higher weight to the RCCA module according to the energy map, which can let each road pixel receive more relation information from the road part. In this way, the geometric information can be considered largely in the attention process, which is the reason why the result of the DCCA module can retain the advantages of the RCCA module.

#### 4.3.3. Example with Both Crisscross Shaped Roads and Rotated Crisscross Shaped Roads

We put an image containing both a rotated crisscross-shaped road and a crisscross-shaped road as an example.

**Figure 26:**
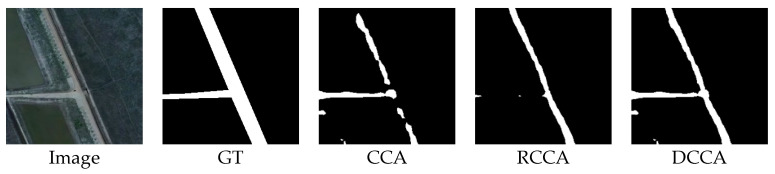
The image, ground truth and the output compilation.

Figure 26 shows that the CCA module has a relatively poor performance when dealing with sloping roads and rotated-crisscross shaped roads. Meanwhile, the RCCA module will miss some road parts when the roads are crisscrossed. The output of the DCCA module complements the advantages and disadvantages of the CCA module and RCCA module. Through the SAF module inside the DCCA module, a larger weight can be assigned to the RCCA module on inclined roads, while a larger weight can be assigned to the CCA module on horizontal and vertical roads to obtain the best results in the end, which is the reason why the DCCA module can adaptively focus on roads of different shapes, and the result is the best.

**Figure 27:**
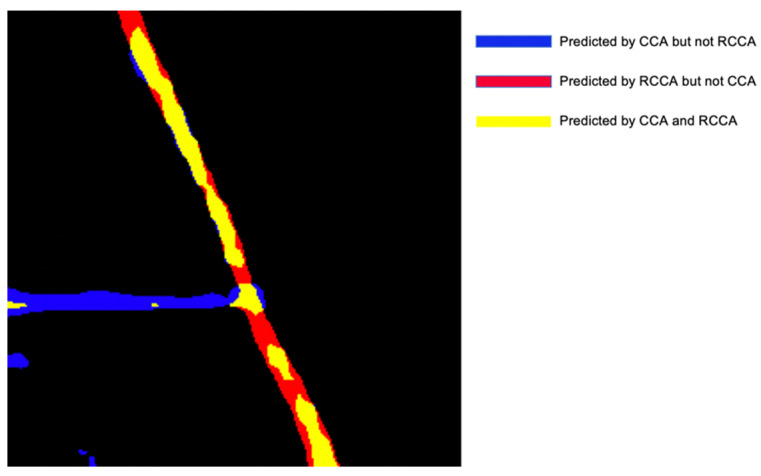
The comparison between CCA module and RCCA module.

In Figure 27, the yellow part is the common prediction of the RCCA and CCA, and the red part is predicted by the RCCA module but not by the CCA module, while the blue module is the opposite. From the comparison of these two modules, we can see clearly that the CCA module plays an important role in the extraction of crisscross-shaped roads, while the RCCA module also has a better result in the extraction of rotated crisscross-shaped roads.

This result shows that the CCA module and RCCA module have their own directional advantages. According to this, the DCCA module can let each module maximize its advantages to obtain better results.

### 4.4. Some Typical Examples

**Figure 28:**
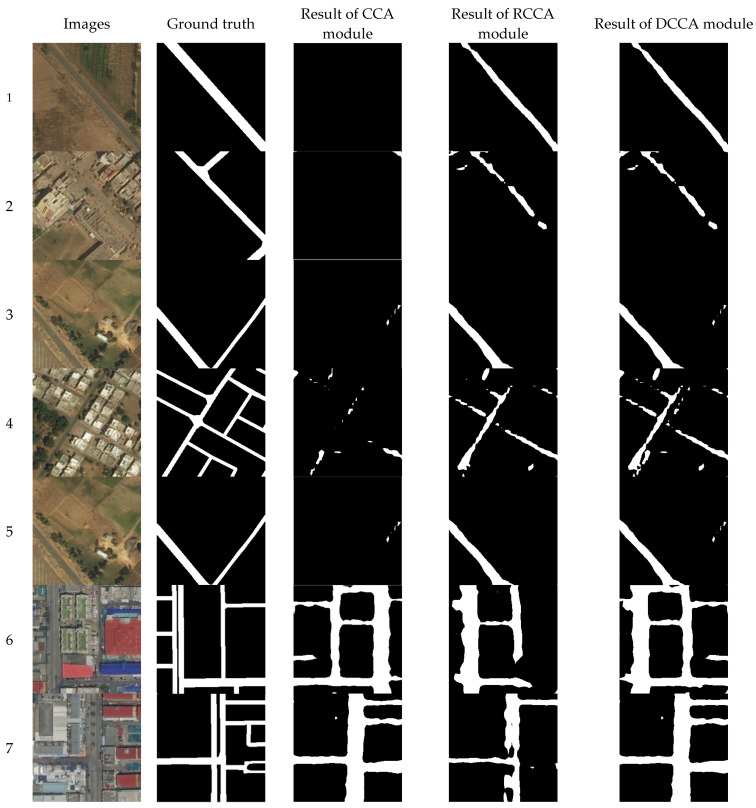

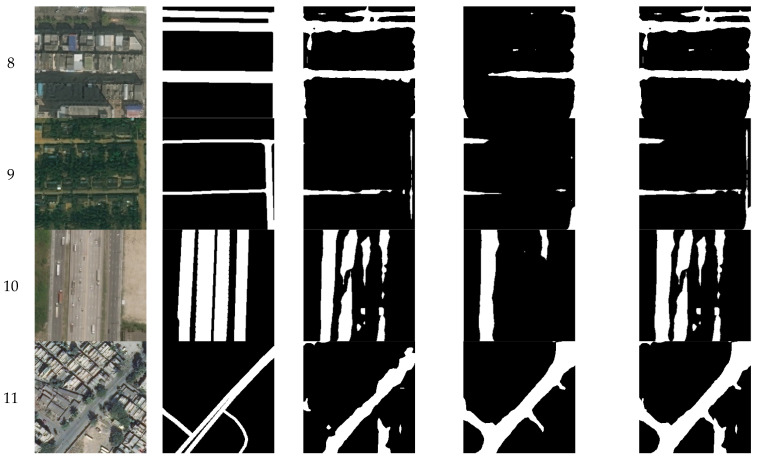
Typical examples.

In Figure 28, the first column are the input images and the figures in the second column are the ground truths. The figures in the third column are the results of the CCA module, while the figures in the fourth column are the results of the RCCA module. The figures in the fifth column are the results of the DCCA module.

In examples 1 to 5, the CCA module cannot extract sloping roads in many cases. However, in these cases, roads can be extracted by the RCCA module. In examples 6 to 10, the output of the RCCA module misses parts of the roads, while the CCA module has a better performance on these crisscross-shaped roads. In these 10 examples, the DCCA module can allow each module to play its own advantage to obtain a better result. This is the reason why the DCCA module can always have the best result. When there are more roads in crisscross shape, the DCCA module will distribute more weights to the CCA module. In contrast, the same situation will come to the RCCA module. Typically, Example 11 contains both crisscross-shaped roads and rotated crisscross-shaped roads. The CCA and RCCA modules make up for each other, and then the result of the DCCA module is the best.

## 5. Conclusions

This paper proposes a special attention mechanism for road extraction, the DCCA module. This module is designed from two attention modules based on different directions, the CCA module and the RCCA module. It also contains the SAF module, a module that can assign weights to the CCA module and RCCA module based on pixel similarity. Since the recurrent dilemma caused by the recurrent mechanism is avoided, the DCCA module can effectively reduce the similarity distortion in the relation between each point pair in the attention process.

In the experiment, the indicators of the DCCA module are 4% higher than those of the CCA and RCCA modules. By analysing specific samples, the DCCA module combines the merits of the CCA module and RCCA module and has better results for road extraction with a richer directionality. In general, the DCCA module has a breakthrough in road extraction by exploiting geometric road directionality information.

In the future, we still have relevant research that needs further exploration. The directionality of the DCCA module is richer than that of the CCA module, but it is still confined to eight directions. Roads in actual situations are often in more than eight directions. There are some circular roads whose direction changes continuously. How to deal with the directional geometric information of these roads is a question worth considering in the future.

## Figures and Tables

**Table 1:** Applying CCA module to different datasets.

Indicators	Complete Dataset	CC Dataset	RCC Dataset
Road IOU	0.4948	0.5442	0.4498
Mean IOU	0.7030	0.7327	0.6759

**Table 2:** Applying RCCA module to different datasets.

Indicators	Complete Dataset	CC Dataset	RCC Dataset
Road IOU	0.5067	0.5242	0.4909
Mean IOU	0.7117	0.7229	0.7015

**Table 3:** Applying DCCA module to different datasets.

Indicators	Complete Dataset	CC Dataset	RCC Dataset
Road IOU	0.5414	0.5722	0.5140
Mean IOU	0.7256	0.7454	0.7077

**Table 4:** The results of the different attention module on complete dataset.

Indicators	CCA	RCCA	DCCA
Road IOU	0.4948	0.5067	0.5414
Mean IOU	0.7030	0.7117	0.7256

**Table 5:** The comparison results of other models.

Models	Road IOU	Mean IOU
DCCA	0.5414	0.7256
DCCA (with 30°)	0.5275	0.7282
Non-local CBAM PSPNet	0.5411 0.5325 0.5357	0.7372 0.7318 0.7322

**Table 6:** The energy comparison of CCA and RCCA sampling.

Classification	CCA	RCCA
Road	10.21	7.96
Non-road	7.95	−0.14

**Table 7:** The energy comparison of CCA and RCCA sampling.

Classification	CCA	RCCA
Road	6.97	5.75
Non-road	1.12	3.43
